# Validating the implementation of an indicator reporting policies and laws on free public maternal health-related services in the era of universal health coverage: A multi-country cross-sectional study

**DOI:** 10.1371/journal.pone.0299249

**Published:** 2024-03-13

**Authors:** Magdalene A. Odikro, Ernest Kenu, Richard M. Adanu, Delia A. B. Bandoh, Mabel Berrueta, Suchandrima Chakraborty, Jewel Gausman, Nizamuddin Khan, Ana Langer, Carolina Nigri, Veronica Pingray, Sowmya Ramesh, Niranjan Saggurti, Paula Vázquez, Caitlin R. Williams, R. Rima Jolivet

**Affiliations:** 1 Department of Epidemiology and Disease Control, University of Ghana School of Public Health, Greater Accra, Ghana; 2 Department of Population, Family, and Reproductive Health, University of Ghana School of Public Health, Accra, Ghana; 3 Institute for Clinical Effectiveness and Health Policy, Buenos Aires, Argentina; 4 Population Council, New Delhi, India; 5 Women and Health Initiative, Department of Global Health and Population, Harvard University T.H. Chan School of Public Health, Boston, Massachusetts, United States of America; 6 Department of Health Science, Kinesiology, and Rehabilitation, Universidad Nacional de La Matanza, Buenos Aires, Argentina; 7 Department of Maternal & Child Health, Gillings School of Global Public Health, University of North Carolina at Chapel Hill, Chapel Hill, North Carolina, United States of America; Public Library of Science, UNITED KINGDOM

## Abstract

**Background:**

The concept of universal health coverage (UHC) encompasses both access to essential health services and freedom from financial harm. The World Health Organization’s Maternal Newborn Child and Adolescent Health (MNCAH) Policy Survey collects data on policies that have the potential to reduce maternal morbidity and mortality. The indicator, “*Are the following health services provided free of charge at point-of-use in the public sector for women of reproductive age*?”, captures the free provision of 13 key categories of maternal health-related services, to measure the success of UHC implementation with respect to maternal health. However, it is unknown whether it provides a valid measure of the provision of free care. Therefore, this study compared free maternal healthcare laws and policies against actual practice in three countries.

**Methods and findings:**

We conducted a cross-sectional study in four districts/provinces in Argentina, Ghana, and India. We performed desk reviews to identify free care laws and policies at the country level and compared those with reports at the global level. We conducted exit interviews with women aged 15–49 years who used a component service or their accompanying persons, as well as with facility chief financial officers or billing administrators, to determine if women had out-of-pocket expenditures associated with accessing services. For designated free services, prevalence of expenditures at the service level for women and reports by financial officers of women ever having expenditures associated with services designated as free were computed. These three sources of data (desk review, surveys of women and administrators) were triangulated, and chi-square analysis was conducted to determine if charges were levied differentially by standard equity stratifiers. Designation of services as free matched what was reported in the MNCAH Policy Survey for Argentina and Ghana. In India, insecticide-treated bed nets and testing and treatment for syphilis were only designated as free for selected populations, differing from the WHO MNCAH Policy Survey. Among 1046, 923, and 1102 women and accompanying persons who were interviewed in Argentina, Ghana, and India, respectively, the highest prevalence of associated expenditures among women who received a component service in each setting was for cesarean section in Argentina (26%, 24/92); family planning in Ghana (78.4%, 69/88); and postnatal maternal care in India (94.4%, 85/90). The highest prevalence of women ever having out of pocket expenditures associated with accessing any free service reported by financial officers was 9.1% (2/22) in Argentina, 64.1% (93/145) in Ghana, and 29.7% (47/158) in India. Across the three countries, self-reports of out of pocket expenditures were significantly associated with district/province and educational status of women. Additionally, wealth quintile in Argentina and age in India were significantly associated with women reporting out of pocket expenditures.

**Conclusions:**

Free care laws were largely accurately reported in the global MNCAH policy database. Notably, we found that women absorbed both direct and indirect costs and made both formal and informal payments for services designated as free. Therefore, the policy indicator does not provide a valid reflection of UHC in the three settings.

## Introduction

Many countries have enacted national policies to adopt universal health coverage (UHC), aligning with the World Health Organization (WHO)’s Sustainable Development Goal 3.8 to achieve UHC in all countries [1–4]. UHC strives to ensure that individuals and communities receive the complete range of essential services from health promotion through prevention, treatment, rehabilitation, and palliative care without financial hardship [1–3]. UHC incorporates two major dimensions: service coverage to promote equitable access to essential services for population health, and protective mechanisms so no person is driven into poverty as a result of seeking healthcare, especially vulnerable populations [1]. These vulnerable populations include pregnant women, adolescents, and children, who often lack the independent means to pay for required healthcare services [5–7]. The two dimensions of UHC are critical to ending preventable maternal mortality for women of reproductive age [8]. Given the priority focus for UHC on free access to maternal health-related services, WHO has developed standardized measurement indicators to monitor countries’ progress toward universally free public maternal healthcare [10, 13].

One of these global policy indicators is, “*Are the following health services provided free of charge at point-of-use in the public sector for women of reproductive age*?” (Table 1). This indicator measures whether 13 maternal health services are provided free to the public for women of reproductive age. Based on its relevance and importance to ensuring UHC, this indicator is a core policy indicator in the Strategies Toward Ending Preventable Maternal Mortality (EPMM) monitoring framework [9, 10]. Additionally, the indicator is one of ten measures validated as part of the multi-country Improving Maternal Health Measurement Capacity and Use (IMHM) Project [11]. Currently, measurement of the indicator uses data from laws and polices collated on the WHO’s Maternal, Newborn, Child and Adolescent Health (MNCAH) Policy Survey from the most current update in 2018 [12, 13]. However, it remains unclear whether this indicator accurately captures the country-level laws and policies or provides a valid reflection of their actual implementation during the provision of maternal health-related care.

**Table 1:** Indicator definition and metadata.

**Indicator name/definition: *Are the following health services provided free of charge at point-of-use in the public sector for women of reproductive age*?**	
• Family planning • Antenatal care • Childbirth (normal) • Cesarean section • Management of other birth complications • Postnatal care for mother • Immunization services during pregnancy • Insecticide-treated bed nets • Pharmaceutical products and/or other medical supplies if required for diagnosis and treatment or childbirth • Testing and treatment for sexually transmitted infections Testing and treatment for syphilis • Testing and treatment for HIV • Screening for cervical cancer	
Response options • Yes, for all women • Yes, for selected population groups • No • Unknown	Indicator reference WHO MNCAH Policy Survey, 2018

Establishing valid indicators is crucial to inform effectiveness of policy. How well the indicator reflects actual policies, i.e., comparison to a gold standard or criterion validity, is important because having free care policies may not be sufficient to ensure progress toward UHC for maternal health [14]. Indeed, such policies may not translate to the intended care at point-of-use [15]. Women may accrue formal and informal charges for services that are reportedly free at the policy level [7, 15, 16]. Charges may be consequent to deficiencies in the health system, including shortage of essential supplies that may force providers to charge for services designated as free [17, 18], or reflect a demand for informal payments [17, 19]. Thus, the indicator may have weakened construct validity, or ability to capture the concept of free care for maternal health services [14]. In addition, there may be a tendency for the poor, illiterate, and less advantaged to be unfairly charged, moving away from rather than toward equity in UHC for maternal health [20–22].

These limitations with policy indicators underscore a need to empirically determine whether this indicator provides a valid measure of both policy and practice related to maternal health UHC, specifically regarding access to essential services and freedom from financial harm. Further, equity is a key aspect of UHC, so it is important to assess whether the indicator is a valid measure of clinical and financial coverage of essential care for all women who need maternal health services. Thus, this multi-country study compared the value of the indicator as reported globally to evidence collected in each country and identified variance that could threaten the measure’s validity. Our findings provide important evidence to support strengthening this indicator.

## Methods

### Study design

We conducted a cross-sectional study using various data collection approaches that involved desk review for secondary data extraction on policies related to provision of free maternal healthcare and surveys for quantitative data collection. We aimed to answer three validation questions:

1. Does the free care law or policy in the country provide all of the categories of services included in the indicator free of charge or fees to users as reported in the WHO MNCAH Policy Survey?
2. For the categories of services that should be free according to the law/policy in the country, is there evidence that women are paying for the services?
3. If evidence demonstrates women are paying for services that are supposed to be free according to the law/policy in the country, is there evidence that user fees are levied in a systematically differential way to women?

### Participants and sampling

The study was conducted in three countries selected for geographic representation: Argentina, Ghana, and India. For uniformity of site selection across the three settings, a composite index of key maternal health indicators as a proxy for health system performance was computed at the subnational level for each country. Based on this index, we selected one state/region in the top and bottom quartiles and one district/province in each state/region in the top and bottom quartiles each (terciles in Argentina due to low population density) for primary data collection (i.e., four districts/provinces across two states/regions in each country). Details on the development of the index and sampling strategy that guided selection of states, districts, and facilities are reported elsewhere [23]. In each country, a list of all public health facilities registered with the government was obtained from the district health department within each study district/province.

We interviewed women aged 15–49 years who accessed any component maternal health service in the specified indicator categories from a public facility in the selected district. For women who underwent a complicated birth or cesarean section, we identified whether they were accompanied by a companion during birth and invited the companion to participate. We excluded women who were unable or declined to answer survey questions.

For each of the 13 services in Ghana and India and applicable 12 services in Argentina, we interviewed a minimum of 20 women in each study district. Insecticide-treated nets were not applicable in Argentina’s country setting. Participants were sampled for one primary component service but were interviewed for all services received that day. We also interviewed the chief financial officer or similar administrator familiar with billing practices in maternal health services where women obtained services. In Ghana, financial officers oversee more than one facility and thus were interviewed once for all facilities within the same management structure.

### Definitions and outcomes

We defined “free of charge” as free of all charges, fees, and related out-of-pocket expenditures, both formal and informal, for women seeking the services included under this indicator, as verified from surveys with women and financial officers.

Our primary outcome measures were:

- Prevalence of out of pocket expenditures made when accessing services designated as free, as reported by women.
- Prevalence of need to charge for services designated as free at the facility level, as reported by financial officers.

For the purpose of this study, based on recommendations from an expert technical consultation, we tabulated the value of this indicator as follows [24]:

- Yes = Yes for all services
- Partial = Yes, for selected services
- No = No for any services
- Unknown

The findings based on this tabulation were then disaggregated by EPMM equity stratifiers (wealth, age, education, geographic region, and rural/urban residence)

We also quantified the reasons why women reported making payments as well as the financial officers’ awareness of the existence of free care laws and their reasons for needing to charge for services.

### Data collection

For the first validation question, data from the most recent WHO MNCAH Policy Survey from 2018 were extracted for each country. We conducted a desk review of national and subnational laws and policies on free care provision for maternal health in each country. In Argentina, we systematically searched the "Sistema Argentino de Información Jurídica" website of the Ministry of Justice and Human Rights for all laws related to the financing of healthcare services and consulted with two local legal experts to ascertain inclusion of all relevant documents and correct interpretation of overlapping legal documents. Ultimately the Constitution of the Nation and Migratory Policy (Law 25,871) were determined to provide the overarching legal framework in the country. In Ghana, we searched internet databases for relevant policies. The Free Maternal Health Care Policy under the National Health Insurance Scheme primarily covers provision of free maternal healthcare in Ghana. Additional documents reviewed were program-level policies from the National Malaria Control Program and the National HIV/AIDS Control Program. Additionally, we consulted maternal health experts in the field to ensure exhaustive review of relevant source documents especially because the free care policy in Ghana was not codified in law. In India, we reviewed the Janani Shishu-Suraksha Karyakaram, which is the national governmental program that covers all aspects of maternal and child health including provision of free care. Additionally, program-level policy documents from the government portal were reviewed for the National Vector Borne Disease Program, National AIDS Control Program, and National Program for Prevention and Control of Cancer, Diabetes, Cardiovascular Diseases and Stroke. A pre-designed, standardized data extraction form was used by each country to collect WHO MNCAH Policy Survey and country-level data. The reference for each policy document backing provision of free service at the country level was also entered in the extraction sheet.

For the second validation question, we interviewed financial officers and women. We considered each chief financial officer as a proxy for the health facilities they represented. In Argentina, eligible financial officers were sent a secure link via email soliciting their consent to participate. Those who gave their consent, received another link to access an online survey. Those who elected to respond via paper-based survey were asked to complete the form in a private room within the facility where they practice; completed paper-based surveys were sealed in an envelope and transferred to the data center. In Ghana, in-person interviews were held with consenting financial officers for each facility in a private setting. In India, consenting financial officers were interviewed via phone. From financial officers, we collected information about facility type, district, location, and maternal health services provided at facility; officers’ demographic information, age and awareness of the existence of free care laws for the 13 services, services for which there is ever need for payment or out-of-pocket expenditures, and reasons for any charges. Financial officer interviews were conducted in June 2020–July 2021 in Argentina, May–September 2020 in Ghana, and July–September 2020 in India.

We also conducted exit interviews with women and healthcare companions. Across the three settings, research staff approached eligible women and their accompanying persons after the women received service. Staff described the study, obtained consent, and conducted the survey in a secured room to ensure confidentiality. Interviews with women were conducted from May-October 2021 in Argentina, January–March 2021 in Ghana, and July–September 2020 in India. From the women, we collected information on specific maternal health services accessed during the current visit, any payments made or out-of-pocket expenditures for the services, the cadres of healthcare providers to whom payments were made, and reasons for payments. In India, where some services were designated free for selected populations, we only interviewed women designated to receive free services.

### Data analysis

All data were entered into REDCap, a password-protected secure web-based platform, and exported to Microsoft Excel 2016 to check for errors and remove duplicates. Clean data were exported to Stata Version 14 for analysis using standard codes written for survey data. To ensure anonymity, we report deidentified districts/provinces.

For the first validation question, we documented the services designated as free for women based on country laws and policies and compared to those reported in the recent WHO MNCAH Policy Survey for each country. For the second validation question, we triangulated findings from the desk review with surveys of women and financial officers. From surveys of women and financial officers, we calculated frequencies and proportions of demographic variables, proportion of women who reported paying for services that are designated free, proportion of facility financial officers reporting charges for free services in their facility, and reasons for payments reported by women. We analyzed each component service based on the responses from women. Findings from the three sources were compared, and variance was determined. Due to sparcity of data, in presenting reasons stated by women and financial officers for charges made, we aggregated multiple responses across the 12 or 13 applicable sevices by country.

Stratification of all maternal health indicators wherever feasible by factors frequently associated with disparities was prioritized during the development of the EPMM monitoring framework, and five minimum standard equity stratifiers were identified during that process [10]. For the third validation question, chi-squared test was used to assess whether the charges to women differed by these standard equity stratifiers from the EPMM Phase II indicator set, including: age, wealth, area of residence (urban/rural), district or province, and educational attainment. Five relative and continuous wealth quintiles were calculated using principal component analysis of survey responses. For differential payments, p = 0.05 was considered statistically significant.

### Ethical considerations

We received institutional review board approval from Harvard University (approval ID: IRB19-1086). The study was approved in Argentina by the Comité de Ética de la Investigación de la Provincia de Jujuy (approval ID not applicable), Comisión Provincial de Investigaciones Biomédicas de la Provincia de Salta (approval ID: 321-284616/2019), Consejo Provincial de Bioética de la Provincia de La Pampa (approval ID not applicable), and Comité de Ética Central de la Provincia de Buenos Aires (approval ID: 2919-2056-2019). In Ghana, the study was approved by the Ghana Health Service Ethical Review Board (approval ID: GHS-ERC022/08/19). In India, the study was approved at the national level by the population council IRB (approval ID: 889) and at the local level from Sigma-IRB (approval ID: 10052/IRB/19-20). Additionally, permission was obtained from all required national and sub-national government health authorities and from facility administrators to carry out the study. Written informed consent was obtained from all study participants before the surveys were administered. The age of majority is 18 years old in Ghana, India, and Argentina; however, Argentine Civil Code states 16 years or older for those making decisions related to the care of their body. We requested parental consent and subject assent for subjects 15–17 years where applicable in Ghana and India, and 15 years old in Argentina. Data were stored using level IV Harvard security measures, including use of Harvard REDCap for data management, locked and secure data storage facilities, and encrypted data storage devices. We blinded the provinces and districts using random numbers in this publication to reduce risk of disclosure of the study sites.

## Results

### Survey demographics

Table 2 displays the characteristics of survey respondents in each country.

**Table 2:** Characteristics of survey respondents.

	Country n (%)		
Characteristics of interviewed women	Argentina	Ghana	India
Women interviewed	1041 (99.5)	923 (100)	1077 (97.7)
Accompanying person responded	5 (0.5)	0 (0)	20 (1.8)
Total number of respondents	1046 (100)	923 (100)	1102 (100)
District, n (%) *			
1	262 (25.0)	249 (26.9)	284 (25.8)
2	249 (23.8)	254 (27.5)	290 (26.3)
3	280 (26.8)	227 (24.6)	261 (23.6)
4	255 (24.3)	193 (20.9)	267 (24.2)
Age, n (%)			
18–24 years	357 (34.3)	357 (40.9)	414 (38.4)
25–34 years	473 (45.4)	403 (46.2)	571 (53.0)
≥35 years	181 (17.4)	112 (12.8)	92 (8.5)
Missing	30 (2.9)	-	-
Literacy level, n (%)			
Read and write	1033 (99.2)	326 (35.2)	957 (88.9)
Read only	4 (0.38)	31 (3.4)	19 (1.8)
Can sign only	2 (0.2)	10 (1.1)	49 (4.5)
Cannot read and write	1 (0.10)	552 (59.8)	52 (4.8)
Refused	–	0.4 (4)	0 (0)
Missing	1 (0.1)	-	-
Highest level of education completed, n (%)			
No formal education	5 (0.5)	339 (36.8)	38 (3.7)
Primary/elementary	243 (23.4)	309 (33.5)	234 (22.8)
Secondary	625 (60.0)	200 (21.7)	481 (46.9)
Technical/vocational	166 (15.9)	19 (2.1)	72 (7.0)
Tertiary/college	–	47 (5.1)	111 (10.8)
Graduate/professional degree	–	4 (0.4)	89 (8.7)
Other	–	4 (0.4)	0 (0)
Missing	2 (0.2)	-	-
Has health insurance, n (%)			
No	763 (73.3)	66 (6.6)	162 (15.0)
Yes	167 (16.0)	860 (93.4)	910 (84.5)
Don’t know	0 (0)	0 (0)	0 (0)
Refused	0 (0)	0 (0)	5 (0.5)
Missing	111 (10.7)	-	-
Wealth quintile, n (%)			
Poorest	72 (6.9)	253 (28.4)	20 (1.9)
Poor	219 (21.0)	207 (23.2)	53 (4.9)
Middle	318 (30.6)	182 (20.4)	129 (11.9)
Rich	254 (24.4)	146 (16.4)	276 (25.6)
Richest	61 (5.9)	103 (11.6)	599 (55.6)
Missing	117 (11.2)	-	-
Type of facility, n (%)			
Primary care	417 (39.9)	875 (94.7)	243 (22.05)
Secondary care	256 (24.5)	48 (5.2)	611 (55.44)
Tertiary care	373 (35.7)	-	248 (22.50)
Service obtained by women, n (%) *			
Family planning	125 (11.9)	85 (9.2)	95 (8.6)
Antenatal care	106 (10.1)	347 (37.6)	135 (12.3)
Childbirth (normal)	95 (9.1)	84 (9.1)	94 (8.5)
Cesarean section	92 (8.8)	44 (4.8)	90 (8.2)
Management of other birth complications	93 (8.9)	86 (9.3)	84 (7.6)
Postnatal care for mother	91 (8.7)	87 (9.4)	90(8.1)
Immunization services during pregnancy	92 (8.8)	167 (18.1)	83 (7.5)
Insecticide-treated bed nets	–	131 (14.2)	89 (8.1)
Pharmaceutical product/other medical supplies required for diagnosis and treatment or childbirth	83 (7.9)	229 (24.8)	85 (7.7)
Testing and treatment of STI	98 (9.4)	97 (10.5)	84 (7.6)
Testing and treatment for syphilis	90 (8.6)	98 (10.6)	81 (7.4)
Testing and treatment for HIV	99 (9.5)	123 (13.3)	89 (8.1)
Screening for cervical cancer	96 (9.2)	0 (0)	88 (7.9)
**Characteristics of interviewed financial officers**			
Number of financial officers/facilities	22	115 /145 ^#^	158
Mean years of working experience ± SD	23.0 (± 9.8)	9.1 ± 7.5	11.2 ± 8
Location			
Urban	22 (100)	37 (32.2)	36 (22.8)
Rural	0 (0)	78 (67.8)	122 (77.2)
Type of facility			
Primary care	13 (59.05)	144 (99.3)	84 (53.2)
Secondary care	5 (22.7)	1 (0.7)	70 (44.3)
Tertiary care	4 (18.18)	0 (0)	4 (2.5)

* Deidentified districts/provinces across study settings.

^#^In Ghana, 115 financial officers were interviewed representing 145 facilities

#### Argentina

We interviewed a total of 1046 women and accompanying persons in Argentina. The sample was almost evenly distributed across the four provinces, and 45.4% were aged 25–34 years. Almost all of the women could read and write (99.2%), 60% had completed secondary education, 73.3% did not have health insurance, and 30.6% were in the middle wealth quintile. The most frequent services obtained were family planning services (11.9%) and antenatal care (10.1%). We interviewed 22 financial officers from urban public facilities in Argentina, 13 of whom (59.1%) were stationed at primary health facilities, with a mean ± SD working experience of 23 ± 9.8 years.

#### Ghana

In Ghana, we interviewed 923 women almost evenly distributed across the districts. Almost half (46.2%) were 25–34 years old, 59.8% could not read and write, 36.8% had no formal education, and 93.4% had health insurance. The sample was evenly distributed among wealth quintiles, with most participants (28.4%) in the poorest quintile. The most frequent services obtained were antenatal care (37.6%) and pharmaceutical and other medical supplies (24.8%). We interviewed 115 financial officers in Ghana representing 145 health facilities, 103 of these facilities (71.0%) were in rural settings and 144 (99.3%) were primary care facilities. Financial officers had a mean ± SD working experience of 9.0 ± 7.5 years.

#### India

In India, we interviewed 1102 women and accompanying persons. The sample was evenly split across the four districts, with 53.0% aged 25–34 years. Most participants (88.9%) could read and write, 46.9% had a secondary-level education, and 84.5% had health insurance. Most participants (55.6%) were in the richest quintile. Services were well-distributed in India, with the most participants receiving antenatal care (12.3%) and family planning (8.6%). We interviewed a total of 158 financial officers, 77.2% of whom worked in rural facilities and 53.2% in primary care facilities, with mean ± SD working experience of 11.2 ± 8 years.

### Concurrence between country-level policy and MNCAH policy survey

Table 3 compares source data from all three countries with reported global data. In Argentina, country-level policies designated 12 out of the 12 applicable services as free. This matched what was reported in the WHO MNCAH Policy Survey. In Ghana, all 13 services were designated as free by country-level policies and the WHO MNCAH Policy Survey. In India, country-level policies designated 11 out of the 13 services as free for all women; insecticide-treated bed nets and testing and treatment for syphilis were free for selected populations (those living in areas where vector-borne illnesses and syphilis were endemic). However, the WHO MNCAH Policy Survey designated all 13 services as free.

**Table 3:** Validation of results from policy review with interviews of women and financial officers.

Service	Argentina					Ghana					India				
	Policy	Women and Accompanying Persons		FOs (N = 22)		Policy	Women		FOs (N = 145)		Policy	Women and Accompanying Persons		FOs (N = 158)	
	Existence of free care laws	Number received service,n	Women report been charged,n (%)	Awareness of free care laws,n (%)	FOs report charging, n (%)	Existence of free care laws	Number received service,n	Women report been charged, n (%)	Awareness of free care laws,n (%)	FOs report charging, n(%)	Existence of free care laws	Number received service, n	Women report been charged, n (%)	Awareness of free care laws, n (%)	FOs report charging, n (%)
Family planning	Y	125	17 (13.6)	22 (100)	2 (9.1)	Y	88	69 (78.4)	56 (38.7)	93 (64.1)	Y	95	70 (73.7)	158 (100)	32 (20.1)
Childbirth (normal delivery)	Y	95	21 (22.1)	15 (68.2)	1 (4.6)	Y	88	35 (39.8)	91 (62.8)	46 (31.7)	Y	94	84 (89.4)	154 (97.4)	46 (29.1)
Cesarean delivery	Y	92	24 (26)	13 (59.1)	1 (4.6)	Y	44	0 (0)	16 (11.0)	11 (7.6)	Y	90	82 (91.1)	140 (88.6)	46 (29.1)
Management of other birth complications	Y	93	8 (8.6)	16 (72.7)	1 (4.6)	Y	91	33 (36.3)	88 (60.7)	19 (13.1)	Y	84	67 (79.8)	147 (93.0)	28 (17.7)
Pharmaceutical products/medical supplies needed for diagnosis and treatment in pregnancy and childbirth	Y	83	15 (18.1)	16 (72.7)	1 (4.6)	Y	250	60 (24)	95 (65.2)	44 (30.3)	Y	85	53 (62.5)	158 (100)	21 (13.3)
Postnatal care for mother	Y	91	13 (14.3)	22 (100)	1 (4.6)	Y	104	9 (8.7)	122 (84.1)	1 (0.69)	Y	90	85 (94.4)	155 (98.1)	43 (27.2)
Antenatal care	Y	106	25 (23.6)	22 (100)	2 (9.1)	Y	383	101 (26.4)	116 (80.0)	16 (11.1)	Y	135	118 (87.4)	157 (99.4)	23 (14.6)
Immunization services during pregnancy	Y	92	11 (11.9)	22 (100)	1 (4.6)	Y	188	1 (0.5)	123 (84.8)	1 (0.69)	Y	83	51 (61.5)	158 (100)	42 (26.6)
Insecticide-treated bed nets distributed during pregnancy (where relevant)	N/A	N/A	N/A	N/A	N/A	Y	150	4 (2.7)	137 (94.5)	0 (0)	Y*	89	54 (60.7)	153 (96.8)	1 (6.3)
Testing and treatment for sexually transmitted infections	Y	98	12 (12.2)	22 (100)	1 (4.6)	Y	117	57 (48.7)	88 (60.8)	29(20.0)	Y	84	66 (78.6)	155 (98.1)	12 (7.6)
Testing and treatment for syphilis	Y	90	19 (21.1)	22 (100)	1 (4.6)	Y	119	53 (44.5)	78 (53.8)	20 (13.8)	Y*	81	63 (77.8)	157 (99.4)	39 (24.7)
Testing and treatment for HIV	Y	99	12 (12.1)	22 (100)	1 (4.6)	Y	147	50 (34)	127 (87.6)	2 (1.38)	Y	89	62 (69.9)	158 (100)	47 (29.7)
Screening for cervical cancer	Y	96	10 (10.4)	21 (95.4)	1 (4.6)	Y	0	0 (0)	10 (6.9)	17 (11.7)	Y	88	79 (89.8)	154 (97.4)	14 (8.9)

FO: financial officer

*Free for selected populations. Red color indicates discordance with WHO MNCAH Policy Survey.

### Payments associated with maternal health services designated as free by policy

Table 3 also displays data on payments made for services desginated as free in all three countries and Table 4 displays the reasons provided for payments. In Argentina, financial officers reported high awareness of free care laws for the 12 services, ranging 59.1%–100%. Officers also reported a low prevalence of women seeking care in their facilities ever needing to make out-of-pocket payments associated with the 12 free services, ranging from 4.6% for maternal immunization services, childbirth (vaginal and cesarean birth), management of birth complications, pharmaceutical products/other supplies for childbirth, postnatal care, testing and treatment of STIs/syphilis/HIV, and cervical cancer screening to 9.1% for family planning and antenatal care. The reasons they reported for charges were mainly for purchase of supplies (13.7%), registration fees (13.7%), and laboratory fees (13.7%).

**Table 4:** Reasons for payments associated with services designated as free, countries.

	Country, n (%)					
	Argentina		Ghana		India	
Reason for charges	Women	FOs	Women	FOs	Women	FOs
Total Number of Responses	238	102	490	256	2539	721
Purchase supplies	61 (25.6)	14 (13.7)	194 (39.6)	123 (48.1)	200 (7.9)	87 (12.1)
Pay healthcare worker	0 (0)	12 (11.8)	3 (0.6)	5 (1.95)	242 (9.5)	0 (0)
Pay fee for service	4 (1.7)	12 (11.8)	164 (33.5)	70 (27.3)	237 (9.3)	0 (0)
Lab fee	3 (1.3)	14 (13.7)	61 (12.4)	31 (12.1)	154 (6.1)	2 (0.3)
Consultation fee	0 (0)	12 (11.8)	7 (1.4)	5 (1.9)	3 (0.1)	0 (0)
Registration fees	0 (0)	14 (13.7)	11 (2.2)	0 (0)	147 (5.8)	153 (21.2)
Travel costs	136 (57.1)	12 (11.8)	1 (0.2)	0 (0)	833 (32.8)	290 (40.2)
Food costs	34 (14.3)	12 (11.8)	0 (0)	0 (0)	527 (20.8)	189 (26.2)
Other	0 (0)	0 (0)	18 (3.7)	22 (8.6)	191 (7.5)	0 (0)
Don’t know	0 (0)	0 (0)	28 (5.7)	0 (0)	3 (0.1)	0 (0)
Refused	0 (0)	0 (0)	1 (0.2)	0 (0)	2 (0.1)	0 (0)

*Multiple responses were captured for both women and financial officers (FOs).

*Percentages calculated using total number of responses as N ‘s

Women in Argentina reported having costs associated with all 12 services designated as free, with prevalence of associated out of pocket costs ranging from 8.6% for management of other birth complications to 26.0% for cesarean sections. Of the 238 times women in Argentina reported costs associated with services designated as free, 25.6% were payments for purchase of supplies and 57.1% for travel costs.

In Ghana, financial officers reported awareness of free care laws ranging from 6.9% for cervical cancer screening to 94.5% for insecticide-treated bed nets. Officers reported ever needing to charge for 12 out of 13 services (except insecticide-treated bed nets), with a prevalence ranging from 0.69% for postnatal care for mother and immunization services during pregnancy to 64.1% for family planning. Financial officers reported fees were mainly charged for purchase of supplies (48.1%) and services rendered (27.3%).

Women in Ghana reported being charged for 11 out of the 13 services designated as free (cervical cancer screening was not routinely offered in districts, and no woman was charged for cesarean section), with prevalence of fees ranging from 0.5% for immunization services to 78.4% for family planning. Of the 490 times women in Ghana reported being charged for services designated as free, 39.6% were payment for purchase of supplies and 33.5% were payment for services rendered.

In India, financial officers reported awareness of free care laws ranging from 88.6% for cesarean delivery to 100% for testing and treatment of HIV. Officers reported ever charging for all 13 services, with a prevalence ranging from 6.3% for insecticide-treated bed nets to 29.7% for testing and treatment of HIV. Financial officers reported fees charged were mainly for travel costs (40.2%), food costs (26.2%), and registration fees (21.2%).

Women in India frequently reported been charged for all 13 services designated as free, with prevalence of fees ranging 60.7% for insecticide-treated bed nets to 94.4% for postnatal maternal care. Of the 2,539 times women in India reported being charged for services designated as free, 32.8% were payment for travel costs and 20.8% for food costs.

### Payments associated with services designated as free based on women’s demographic characteristics

Table 5 displays the association between payment for services designated as free and women’s characteristics. Women’s out of pocket expenditures when accessing services designated as free varied significantly by various standard EPMM equity stratifiers across the three countries.

**Table 5:** Payment for services designated as free based on women’s social and EPMM equity stratifiers.

Country	Argentina			Ghana			India		
Equity stratifier	Paid, n (%)	Did not pay, n (%)	p	Paid, n (%)	Did not pay, n (%)	p	Paid, n (%)	Did not pay, n (%)	p
Wealth									
Poorest	15 (20.6)	58 (79.4)		106 (41.9)	147 (58.1)	0.309	7 (35)	65 (13)	0.242
Poor	43 (19.5)	178 (80.5)	**0.045**	82 (39.6)	125 (60.4)		18 (32.7)	37 (67.2)	
Middle	43 (13.5)	275 (86.5)		66 (36.3)	116 (63.7)		42 (31.1)	93 (68.9)	
Rich	(30 (11.8)	224 (88.2)		48 (32.9)	98 (67.1)		77 (27.0)	208 (72.9)	
Richer	8 (13.1)	53 (86.9)		34 (33)	69 (67)		145 (23.9)	462 (76.1)	
Missing	26 (21.9)	93 (78.2)							
Residence									
Rural	2 (5.6)	34 (94.4)	0.087	60 (39.7)	91 (60.3)	0.605	16 (23.2)	418 (76.8)	**0.022**
Urban	163 (16.1)	847 (83.8)		288 (37.5)	480 (62.5)		163 (29.2)	395 (70.8)	
District/province									
1	43 (16.4)	219 (83.6)	**<0.001**	136 (54.6)	113 (45.4)	**<0.001**	40 (14.1)	244 (85.9)	**< .001**
2	58 (23.3)	191 (86.7)		73 (37.8)	120 (62.2)		174 (60.0)	116 (40.0)	
3	42 (15.0)	238 (85.0)		53 (23.4)	174 (76.7)		10.73 (28)	233 (89.3)	
4	22 (8.6)	233 (91.4)		88 (34.7)	166 (65.4)		47 (17.6)	220 (82.4)	
Missing	0 (0)	0 (0)							
Educational attainment						**0.011**			**<0.001**
No formal education	2 (40.0)	3 (60.0)	**0.007**	139 (41)	200 (59)		21 (52.5)	19 (47.5)	
Primary education	50 (18.1)	193 (79.4)		96 (31.1)	213 (68.9)		51 (21.4)	187 (78.5)	
Secondary	97 (15.5)	528 (84.5)		88 (44)	112 (56)		115 (23.4)	378 (76.5)	
Higher than secondary	14 (8.4)	152 (91.5)		24 (34.3)	46 (65.7)		77 (27.7)	201 (72.3)	
Missing	2 (28.5)	5 (71.4)							
Age (years)									
18–24	66 (18.5)	291 (81.5)	0.282	142 (39.8)	215 (60.2)	0.747	94 (21.9)	335 (78.1)	**0.019**
25–34	74 (15.6)	399 (84.4)		151 (37.5)	252 (62.5)		172 (29.7)	407 (70.3)	
≥35	22 (11.8)	164 (88.2)		41 (36.6)	71 (63.4)		23 (24.5)	71 (75.5)	
Missing	3 (10.0)	27 (90.0)							

In Argentina, women’s expenditures varied significantly by province (p < 0.001), educational status (p = 0.007), and wealth quintile (p = 0.045). In Ghana, women’s expenditures varied by district (p < 0.001) and educational status (p = 0.011). In India, women’s expenditures varied by residence district (p < 0.001), educational status (p < 0.001), residence (p = 0.022) and age (p = 0.019).

## Discussion

We assessed validity of a global policy indicator on free care for maternal health services, a measure of UHC intended to monitor access to essential public health service without financial burden for all women of reproductive age. In our desk review, in Argentina and Ghana, we found consistency between existing country-level policies and those reported globally in the WHO MNCAH Policy Survey; in India, we found minor discrepancies between country-level policy on record and what was reported in the WHO MNCAH Policy Survey. However, interviews with women and financial officers revealed that women often had out of pocket expenditures associated with accessing services designated as free by policy. Expenditures were reported for direct and indirect costs of care, and included both formal payments reflecting health system deficiencies, and informal payments not acknowledged by the facility management. Additionally, reported payments varied differentially by some of the demographic characteristics reflected in the standard EPMM equity statifiers across the three settings. Our findings echo existing concerns from stakeholders on the construct validity of this indicator in accurately assessing UHC for maternal health [19, 24, 25].

The consistency of global database information for Argentina and Ghana affirms the criterion validity of this indicator in these settings. Countries are required to submit documentation on free care policies to support their responses [13], indicating that the validation process was thorough for Argentina and Ghana. However, for India there was mismatch between information in the global database and country-level findings. As India also submitted documentation of its reference policies, this mismatch may indicate a simple error. WHO has previously reported issues in its validation of countries’ reported policies [13]. The inconsistency could also be due to discrepancy in policy interpretation. It is worthwhile to note that the two services with mismatch were both designated as free only for select populations at the country level. In our study, we interpreted this to mean the services were not free for everyone in the country; however, it is possible that in the global database this was interpreted as free for all who need it (i.e., those living in vector-borne and syphilis endemic areas). Regardless of the reason, the discrepancy in reported policies between the WHO MNCAH Policy Survey and national-level findings for India raises concerns about the indicator’s criterion validity in this setting. Moreover, numerous studies demonstrate that policy-level declarations do not always reflect the reality at the service provision level [7, 16, 26].

Furthermore, our study uncovered gaps between policy and practice. Overall, we found that women had out of pocket expenditures when accessing services designated as free, as established from triangulation of different sources of data. Women who accessed services reported having associated out of pocket expenditures for all 12 component services in Argentina, all 13 component services in India, and for 11 of 13 component services in Ghana. The expenditures reflect both direct and indirect costs associated with the services obtained, including purchase of supplies across all settings, as well as travel and food costs in Argentina and India. Further, our findings suggest charges that were both formal and informal, for example, service and registration fees in Ghana and India and payments to health workers in India. This finding is consistent with previous research in developing countries showing that women are charged both formally and informally for services designated as free [7, 15, 16]. Although financial officers reported their awareness that the services were free by policy, they also reported that necessity may dictate the existence of women’s out of pocket expenditures. A major reason for this need to charge was for purchase of supplies, in line with previous findings indicating that shortages of essential supplies may force providers to charge for services that should be free [17, 18]. These findings compromise the validity of the indicator as a measure of free universal access to essential maternal health services by reproductive-age women without financial burden in all our study settings.

Generally, few financial officers reported there ever being a need for women to make out of pocket payments when accessing free services. This may be because financial officers feel they are violating policies by charging and thus are less likely to freely report the charges. However, where financial officers did report ever needing to charge for free services, their responses were generally aligned with women’s reports, except in the case of direct payments to healthworkers or fees for services, which likely reflect informal payments. This reiterates the need to ensure an enabling environment for implementation of free care laws [27–29].

We evaluated the validity of the measure by collecting data directly from end-users to corroborate information reported at the policy level—our results suggest that both health system administrators’ and women’s experiences are critical to ensuring the construct validity of the policy indicator, that it captures the intended concept of universal health coverage for maternal health fully, accurately, and reliably from both the supply and demand side. Indeed, other studies have called for changes in data sources and recommended measurement indicators evolve over time to reflect changing policy priorities [30, 31]. Using both end-user data sources also captured the range of formal and informal charges for services, which is vital to help target interventions and policy refinements where necessary.

Another way we tested the indicator’s validity was to change the estimation method. We assessed specific service categories, while original computation of the indicator used categories of women who were charged for free services to assess whether care was free for all, some, or no types of women. Our approach assessed free care by service category and further analyzed findings to determine equity. We chose assessment at the service level because some services are more likely to be charged in certain settings and may have greater costs compared to others. As demonstrated from our findings, the specific services women were charged for differed across the three settings. Evidence shows that targeting interventions to specific populations is less effective than universal coverage of services with human rights implications [32, 33]—it is more beneficial for a particular number of services to be free for all rather than for all services to be free for targeted groups only.

Collecting data at the service level enables disaggregation by individual-level equity factors. Our results showed that services were differentially charged to women based on wealth quintile, district/province, education status, residence, and age within the three settings. Differential charges indicate inequity, with major implications for UHC [16, 26]. Although not directly assessing charges or user fees, previous studies have established inequities in access and utilization of maternal and child health services, with some related to rural–urban differences in costs associated with accessing services in Ghana, Malawi, India, and South Africa [20, 22, 34]. Other studies in Ghana and Burkina Faso have established equity concerns for accessing maternal health services [19, 35, 36]. As equity is an important aspect of UHC, these findings challenge the validity of the indicator as a measure of this construct.

Another concern that challenges the validity of the indicator is the inability of the current measurement to capture all dimensions of UHC. In our study, we captured all costs associated with accessing maternal healthcare by women (formal, informal, direct and indirect costs). Capturing other costs is directly in line with the second dimension of UHC which looks at protective mechanisms so no person is driven into poverty as a result of seeking healthcare [1]. The current measurement of this indicator from the policy level is unable to capture this dimension of UHC because existence and even full implementation of laws on free care only ensure women will not be charged at point of use. Meanwhile, women will continue to suffer financial harm in accessing services due to need to cover associated costs including transportation and food costs as demonstrated by our findings. This further challenges the construct validity of this indicator in accurately measuring UHC for maternal health.

A major strength of this study is our rigorous approach to validate the indicator. Triangulating findings from country-level policies with interviews of women and financial officers and collecting data directly from end-users provided a fuller account of the reality of payments associated with free care, identifying threats to validity in the current measurement of the indicator. Our findings suggest that this policy indicator may need to be complemented by measures that monitor implementation of the policy at the point-of-use by service users (demand side) and at the service delivery level (supply side) for optimal construct validity.

This study also has important limitations. Our findings from self-reported key variables could possibly be affected by courtesy bias, especially in the case of interviews with women. Given that interviewed women were likely to continue receiving services at the same facilities, they may have under-reported charges [37]. Nonetheless, our findings showed significant charges levied to women, even if underreported. In Argentina, due to the inability of women to discern all factors contributing to the categorization of wealth, there were some missing values for the wealth index. However, since this affected all provinces, the effect is unlikely to significantly change our findings in this setting. Further, the data collection method in each country varied for women and financial officers (in-person, telephone, or online interviews), which could have resulted in some information bias. Due to the COVID-19 pandemic, routine service provision was altered and for some services we could not interview women because the services were not offered during the pandemic (e.g., screening for cervical cancer in Ghana) which could have introduced some information bias. Additionally, some districts did not offer some services, limiting the service sample in those districts. Our findings on equity could also be possibly affected by systematic differences in self reporting of some costs due to price sensitivity (eg; transportation costs). For example, it is likely that all (or nearly all) women would have had to pay for transport to the hospital as few live within walking distance. However, only a portion of the sample reported transit costs. It may be that the poorest women are most likely to register this as a cost due to higher price sensitivity. As we are unable to differentiate between these from our data, this limitation should be considered in conducting needed further studies to operationalize data collection for routine measurement of an indicator that tracks implementation of free care at the service level.

## Conclusion

Our results showed that the global WHO MNCAH Policy Survey largely accurately reported free care laws and policies. Nevertheless, we found that women had out of pocket expenditures that reflect both formal and informal charges and bore both direct and indirect costs associated with services designated as free by country-specific policies. Therefore, our results indicate the policy indicator of free care for maternal health services did not provide a valid reflection of UHC in India, Ghana, and Argentina.
